# 固相萃取-超高效液相色谱-串联质谱法检测婴幼儿奶粉中的7种链格孢霉毒素

**DOI:** 10.3724/SP.J.1123.2021.05023

**Published:** 2022-02-08

**Authors:** Jiali XING, Zigeng ZHANG, Ruihang ZHENG, Xiaorong XU, Lingyan MAO, Hai CHENG, Jian SHEN

**Affiliations:** 1.宁波市产品食品质量检验研究院(宁波市纤维检验所), 浙江 宁波 315048; 1. Ningbo Academy of Product and Food Quality Inspection (Ningbo Fibre Inspection Institute), Ningbo 315048, China; 2.中国计量大学生命科学学院, 浙江 杭州 310018; 2. College of Life Sciences, China Jiliang University, Hangzhou 310018, China

**Keywords:** 固相萃取, 超高效液相色谱-串联质谱法, 链格孢霉毒素, 婴幼儿奶粉, solid phase extraction (SPE), ultra-performance liquid chromatography-tandem mass spectrometry (UPLC-MS/MS), *Alternaria* toxins (*A*Ts), infant milk powder

## Abstract

婴幼儿奶粉配料中的植物油易受到链格孢霉菌污染,因而链格孢霉毒素(*A*Ts)成为该类食品的重点检测对象。该研究建立了超高效液相色谱-串联质谱法快速检测婴幼儿奶粉中链格孢酚、链孢酚单甲醚、交链孢霉烯、细交链孢菌酮酸、腾毒素、交链孢毒素Ⅰ、细格菌素7种*A*Ts的方法。通过参数优化确定最佳的质谱与色谱条件,选取BEH-C_18_色谱柱,以0.1%甲酸水溶液-乙腈为流动相,然后分别对提取条件(提取剂比例及提取方式)以及固相萃取条件(萃取小柱、洗脱液种类及体积、上样液pH)进行优化,确定使用乙腈-水(84:16, v/v)为前两次提取剂,乙腈-甲醇-水(45:10:45, v/v/v)为第三次提取剂,水平摇匀30 min为最佳提取方式,以pH 5.5的一级水复溶上样,经HLB小柱净化,10 mL甲醇洗脱,不浓缩直接过0.22 μm滤膜后直接进行色谱分离,色谱分离后以电喷雾正负离子交替多反应监测模式分析。在最佳优化分析条件下,7种*A*Ts在0.5~200 μg/L范围内线性关系良好,判定系数(*R*^2^)>0.9903,检出限为0.15~0.64 μg/kg,定量限为0.54~2.24 μg/kg。在3个不同加标水平下,7种*A*Ts的平均回收率为79.1%~114.3%, RSD≤8.87%。将该方法用于60份实际婴幼儿奶粉样品的测定分析,结果显示一段奶粉和二段奶粉中未发现毒素;三段奶粉中只有1份样品被检出,检出毒素为腾毒素,其含量为4.97 μg/kg。该方法准确、快速、简便、灵敏度高,重复性与稳定性良好,可用于婴幼儿奶粉7种*A*Ts的实际测定。

## Abstract (en)

*Alternaria* toxin is a general term for a class of toxic metabolites produced by *Alternaria*, which widely exists in soil, grain, vegetables, and fruits. This mycotoxin is extremely harmful to human health. It is well known that infant milk powder containing vegetable oil is easily contaminated by *Alternaria* alternata. *Alternaria* toxins have thus become an increasingly important focus in food. Rapid and accurate detection of *Alternaria* toxin residues in food is of great significance for food safety. This requires pretreatment to purify the target toxins and maximize the accuracy and precision of the analysis. In this study, a rapid method based on online solid phase extraction/purification and ultra-performance liquid chromatography-tandem mass spectrometry (online SPE UPLC MS/MS) was established to detect seven *Alternaria* toxins (alternariol monomethyl ether, altenuene, tenuazonic acid, alternariol, tentoxin, altenusin, and altertoxin Ⅰ) in infant milk powder. First, the mass spectrometry and chromatographic conditions were optimized. A BEH-C_18_ column (50 mm×2.1 mm, 1.7 μm) was selected, with 0.1% formic acid aqueous solution-acetonitrile as the mobile phase. Then, the extraction conditions (extraction agent ratio and extraction method) and the solid phase extraction process (extraction column, type and volume of the eluent, and pH of the sample loading solution) were optimized. One gram of milk powder (accurate to 0.01 g) was weighed into a 50 mL tip and bottom plug centrifuge tube. Acetonitrile-water (84:16, v/v) was set as the extraction agent for the first two cycles, and acetonitrile-methanol-water (45:10:45, v/v/v) was set as the third extraction agent. Horizontal shaking for 30 min was the best extraction method. The sample was centrifuged at 9500 r/min for 10 min, and the supernatant extracted many times was mixed and blown with nitrogen at 40 ℃. The sample was redissolved in first-order water (pH 5.5), purified on an HLB column, and successively activated with 6 mL methanol and 6 mL first-order water (pH 5.5). The solution was then loaded onto the column, and the SPE was adjusted to ensure that the water sample flowed through the column at the rate of 1 mL/min so that the column did not dry up during the analysis process before the end of sample loading. The column was rinsed with 12 mL of first-order water. After leaching, the negative pressure filtration was continued for approximately 5 min, followed by elution with 10 mL methanol, and the eluted solution was directly tested after passing through a 0.22 μm filter membrane, without concentration. The analytes were determined by electrospray ionization (ESI) with alternating positive and negative ions. Under the optimal analysis conditions, the linear relationships of the seven *Alternaria* toxins were good in the mass concentration range of 0.5-200 μg/L, with coefficients of determination (*R*^2^)>0.9903. The limits of detection and limits of quantification were 0.15-0.64 μg/kg and 0.54-2.24 μg/kg, respectively. The recoveries of the seven *Alternaria* toxins were 79.1%-114.3%, and the relative standard deviations were less than 8.87% at different concentrations. The method was applied to the determination and analysis of 60 samples of infant milk powder, and the results showed that no toxin was found in stage one or stage two of the milk powder. Only one sample of the stage three of milk powder was detected, which was tentoxin, and the content was 4.97 μg/kg. The developed method is accurate, rapid, simple, sensitive, repeatable, and stable. It can be used for the practical determination of seven *Alternaria* toxins in infant milk powder.

链格孢霉菌广泛存在于泥土和各种农作物里,是污染谷物、油籽与果蔬的一种极为常见的条件致病菌和腐生丝状真菌^[1]^。由于该菌能在潮湿、低温的环境下生长繁殖,环境适应能力较强,可以产生大量的次级代谢产物,这类代谢产物统称链格孢霉毒素(*Alternaria* toxins, *A*Ts),因此即使是冷藏运输的农产品也可能受到*A*Ts的污染^[2]^。现今已有诸多研究表明*A*Ts对人与动物均具有急慢性毒性以及三致效应(致癌、致畸、致突变)等风险存在,对人畜的健康造成了损害。研究表明约有70余种*A*Ts具有明显毒性,欧洲食品安全局(European Food Safety Authority, EFSA)在2011年对*A*Ts的结构进行了细致的描述^[3]^,此外在Georg等^[4]^的研究中可知,链格孢霉毒素由于结构的不同,大体上可分为6种不同的类型:(1)二苯并吡喃酮类及其衍生物,主要毒素包括交链孢烯(altenuene, ALT)、交链格孢酚单甲醚(alternariol monomethyl ether, AME)和链格孢酚(alternariol, AOH); (2)四氨基酸衍生物类,代表毒素为细交链格孢菌酮酸(tenuazonic acid, TeA)和异细交链孢菌酮酸(iso-TeA); (3)二萘嵌苯醌类及其衍生物,包括交链孢毒素Ⅰ(altertoxin Ⅰ, ATX-Ⅰ)、ATX-Ⅱ、ATX-Ⅲ等一类衍生物毒素,是少数链格孢霉的代谢物;(4)丙三羧酸酯类化合物,是一系列长链氨基多元醇,即互隔交链孢霉(Alternaria alternata,AAL)毒素,又可分为AAL-TA、AAL-TB、AAL-TC、AAL-TD和AAL-TE几大类,其中AAL-TA的毒性最强;(5)包括腾毒素(tentoxin, Ten)在内的环形四肽结构;(6)其他结构:细格菌素(altenusin, ALS)等。近年来,与*A*Ts相关的食品安全事件时常发生^[5]^,婴幼儿是受全社会关注的一种特殊群体,而婴幼儿奶粉是有益于婴幼儿生长发育的首选食品。植物油在婴幼儿奶粉中占比很高,又因其易被链格孢霉菌污染,因此对婴幼儿的健康产生了威胁^[6]^。目前已报道的关于*A*Ts检测的主要基质为柑橘^[7]^、番茄^[8]^、苹果^[9]^、麦芯粉^[10]^、啤酒^[11]^、豌豆^[12]^、向日葵籽^[13]^等,且同时检测的*A*Ts种类也不超过6种,关于婴幼儿奶粉中*A*Ts的检测方法相对较少,因此建立婴幼儿奶粉中典型*A*Ts的检测方法具有重要的意义。

目前我国有关*A*Ts的标准只有1项行业标准^[14]^,并且该标准仅涉及4种*A*Ts,应用范围只限于部分果蔬,尚不涵盖婴幼儿奶粉中*A*Ts的检测。现有研究涉及的*A*Ts的检测技术主要有薄层色谱法^[15]^、酶联免疫吸附法^[16]^、气相色谱(GC)^[17]^以及气相色谱-串联质谱法(GC-MS/MS)^[18]^、液相色谱(LC)^[19]^和液相色谱-串联质谱法(LC-MS/MS)^[20]^等。薄层色谱法灵敏度较低,实验操作繁琐且重复性较差;GC和GC-MS/MS经常用于分离和检测易挥发和热稳定的毒素^[21]^,因为*A*Ts相对来说较为稳定且挥发性较差,所以GC和GC-MS/MS在*A*Ts检测方面的应用受到了限制;LC-MS/MS适用性非常广泛,因为其同时具备质谱的高灵敏度与LC的高分离性,能够对*A*Ts准确地定量定性,可以补充LC技术的不足^[22]^。考虑到奶粉基质组成十分复杂,不利于提取,所以选取固相萃取前处理技术净化*A*Ts,可以更有效地将干扰组分和分析物分离^[23]^。基于此,本研究拟开发一种固相萃取结合超高效液相色谱-串联质谱技术(UPLC-MS/MS)同时准确检测婴幼儿奶粉中7种*A*Ts的分析方法。

## 1 实验部分

### 1.1 材料与试剂

从国内各大企业和当地市场收集了60份婴幼儿奶粉样本,其中一段(0~6月龄)、二段(6~12月龄)、三段(12~36月龄)各20份,密封,-20 ℃保存使用。

*A*Ts标准品:AOH、AME、ALT、Ten、TeA、ALS、ATX-Ⅰ,购自上海安谱实验科技股份有限公司,纯度均大于98%;甲醇、乙腈、甲酸均为色谱纯,购自德国Merck公司。

### 1.2 仪器与设备

ACQUITY UPLC I-CLASS超高效液相色谱-Waters XeVO TQ-XS串联质谱仪,配有电喷雾离子源(美国Waters公司); TGL-20M高速台式冷冻离心机(上海卢湘仪离心机仪器有限公司); Vortex 3自动漩涡混合器(德国IKA公司); Milli-Q型超纯水机,电阻率为18.2 MΩ·cm(美国Millipore公司); KS-300EI超声波清洗机(宁波科生设备有限公司); SW22振荡水浴锅(德国Julabo公司); AH-30全自动均质器(睿科仪器有限公司); 0.22 μm有机滤膜(北京捷盛依科科技有限公司); ME204E电子天平(实际分度值为0.0001 g)和FE28 pH酸度计(上海梅特勒-托利多仪器有限公司); N-EVAP112氮吹仪(上海庆开实验设备有限公司); HLB小柱6 mL(上海安谱实验科技股份有限公司)。

### 1.3 UPLC-MS/MS条件

色谱条件:BEH C_18_色谱柱(50 mm×2.1 mm, 1.7 μm),柱温为40 ℃;流动相体系为(A)0.1%(v/v)甲酸水溶液和(B)乙腈;梯度洗脱程序:0~5.0 min, 10%B~95%B; 5.0~7.0 min, 95%B; 7.0~7.5 min, 95%~10%B; 7.5~10.0 min, 10%B。流速:0.4 mL/min;进样量:3 μL。

质谱条件:采用多反应监测(MRM)模式对7种*A*Ts进行分析;数据采集和处理采用MassLynx^TM^ 4.2软件完成;离子源参数为:正负离子开关扫描;毛细管电压1.08 kV;射频透镜1(RF lens 1)和RF lens 2的电压均为15.0 V;离子源温度150 ℃;脱溶剂温度600 ℃;脱溶剂气体流量1000 L/h;锥孔反吹气流量150 L/h,其他质谱参数见表1。

**表 1:** 7种链格孢霉毒素的质谱测定参数

Toxin	Abbreviation	Ionization mode	Parent ion (m/z)	Daughter ion (m/z)	Dwell time/s	Cone voltage/V	Collision energy/eV
Tentoxin	Ten	ESI^+^	415.4	199.2^*^	0.012	25	13
				171.2			18
Alternariol monomethyl ether	AME	ESI^+^	273.2	258.2	0.012	25	25
				128.1^*^			40
Alternariol	AOH	ESI^+^	259.2	213.2	0.012	25	25
				185.1^*^			30
Tenuazonic acid	TeA	ESI^+^	198.2	125.1^*^	0.012	25	15
				153.1			12
Altenuene	ALT	ESI^+^	293.2	257.2^*^	0.012	25	12
				275.4			8
Altenusin	ALS	ESI^+^	291.2	255.2	0.012	25	18
				199.2^*^			30
Altertoxin Ⅰ	ATX-Ⅰ	ESI^-^	351.3	315.2^*^	0.0.12	25	8
				333.3			10

* Quantitative ion.

### 1.4 标准溶液的制备

标准储备液:分别准确称量TeA、AME、AOH、Ten、ALT、ALS和ATX-Ⅰ标准品0.001 g溶于10 mL乙腈中,配制成100 mg/L的标准混合液,取100 mg/L的标准混合液100 μL用乙腈溶解并定容至10 mL,得到1 mg/L的混合标准储备液,密封后置于-20 ℃保存。

标准工作液:用乙腈-水(1∶1, v/v)溶液将标准储备液逐级稀释配制成0.5、1、2、5、10、20、50、100、200 μg/L的7种*A*Ts的混合标准溶液。

基质标准工作液:用空白基质溶液逐级稀释1 mg/L的混合标准储备液,制备成0.5、1、2、5、10、20、50、100与200 μg/L的基质标准工作液。

### 1.5 链格孢霉毒素的提取

分别称取奶粉1 g(精确到0.01 g)于50 mL尖底具塞离心管内,加入标准储备液50 μL,配制成待测奶粉样品。加入15 mL乙腈-水(84∶16, v/v)溶液,加入0.3 mL甲酸,水平摇匀30 min,在9500 r/min下离心10 min,取上清液,沉淀重复提取3次。最后一次提取时加入15 mL乙腈-甲醇-水(45∶10∶45, v/v/v)和0.3 mL甲酸,水平摇匀30 min, 9500 r/min下离心10 min,多次提取的上清液混合后于40 ℃氮吹,之后重悬于12 mL一级水(pH 5.5)中。

### 1.6 链格孢霉毒素的富集

用HLB固相萃取小柱萃取,依次用6 mL甲醇和6 mL一级水(pH 5.5)活化,之后将待测液加入小柱中,调整固相萃取仪,确保水样以1 mL/min的速度流过柱子,在上样结束之前要确保整个过程小柱都不能干,用12 mL一级水淋洗小柱,淋洗结束后继续负压抽滤5 min左右,最后用10 mL甲醇洗脱,振荡混匀1 min,取1 mL混匀液过0.22 μm有机滤膜,待测。

## 2 结果与讨论

### 2.1 仪器条件的优化

2.1.1 色谱条件的优化

在实际测定过程中,色谱柱的选择对目标物的分离、识别与检测十分重要,因为BEH C_18_具有较低的次级相互作用,pH承受能力强,柱效更高,峰形更好^[24]^,因此采用BEH C_18_(50 mm×2.1 mm, 1.7 μm)分离7种*A*Ts。

水-甲醇和水-乙腈都是UPLC-MS/MS常用的流动相体系^[25]^,而且甲酸和甲酸铵的引入一般可以增强靶向响应并改善靶峰^[26]^。在我们之前的研究中,甲酸、乙腈被用作萃取剂,为了保持一致性,本实验采用了3种流动相体系:水-乙腈、0.1%甲酸水溶液-乙腈、0.1%甲酸水溶液(含0.01 mol/L甲酸铵)-乙腈进行了比较。结果表明,甲酸的引入增强了7种靶向*A*Ts的反应;而甲酸铵的引入使靶向TeA的反应减弱,并出现峰形拖尾,因此,选择0.1%甲酸水溶液-乙腈作为流动相体系。

由于7种*A*Ts极性的不同,而且极性相差较大,如ALT的极性特别强,AME的极性相对很弱^[20]^,为了兼顾7种毒素可以同时出现良好的峰形,流动相需要有较大的梯度变化,本实验通过不断调整,选取最佳的洗脱顺序,可以同时获得不同*A*Ts的灵敏度和稳定性。因为ESI扫描检测需要流动相流速较低,所以设置流速为0.4 mL/min。7种*A*Ts几乎都在40 ℃左右稳定^[27]^,不会发生反应,因此,最终选取柱温为40 ℃。

结果显示,优化条件下各种*A*Ts分离效果明显,峰形较好(见图1)。

**图1:**
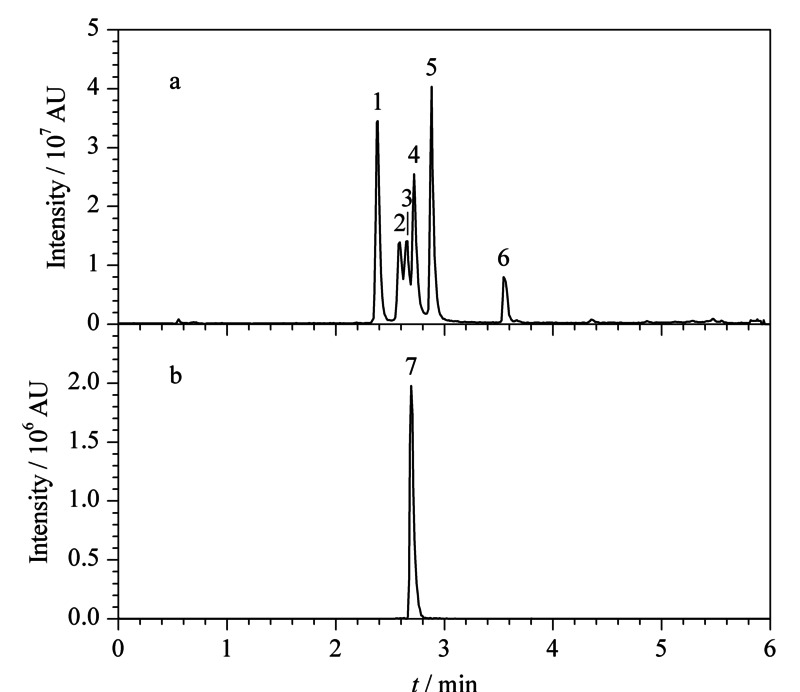
优化条件下(a)6种链格孢霉毒素的正离子图和(b)ATX-Ⅰ的负离子图

2.1.2 质谱条件的优化

因为多反应监测模式可以相当大程度地排除外界干扰,提高信噪比,有助于对目标化合物的定量分析,所以本研究选取多反应监测扫描,并对7种*A*Ts在ESI^+^和ESI^-^模式扫描时进行优化,结果发现ATX-Ⅰ在ESI^-^模式扫描时响应值较高,其他*A*Ts在ESI^+^模式扫描时响应值高,因此选取正负离子交替扫描。锥孔电压、碰撞电压、离子源温度、脱溶剂气体温度、碰撞气体流量和每种毒素的定性、定量离子通过流动注射泵的连续进样进行测定,优化质谱条件,达到每种靶物质的最佳电离效率。分别用ESI^+^和ESI^-^模式对毒素进行扫描,寻找响应值较高的母体离子。进一步改变碰撞电压,进行二次质谱扫描,寻找信号强、稳定性好的子离子。

### 2.2 提取溶剂比例的优化

由于婴幼儿奶粉结构复杂,相对来说难以处理,多次提取有利于增加*A*Ts的回收率^[28]^。而且TeA的酸性和极性较强,因此将提取剂酸化有利于提高TeA的提取率^[29,30]^。本研究根据相关文献^[31]^报道考察了前两次提取剂,即乙腈-水(100∶0, 50∶50, 84∶16, v/v)溶液以及第三次提取中乙腈-甲醇-水(50∶25∶25, 45∶10∶45, v/v/v)溶液(加入甲醇是因为其有利于苯醌类*A*Ts的提取^[32]^)。实验结果如图2a所示,当采用乙腈-水(50∶50, v/v)时,提取液过于混浊,离心不成形,回收率过低;采用纯乙腈提取时,AOH和ALS的回收率相对较低,两种毒素的回收率仅在50%左右,由此可知,水的体积比例不可过高或过低,适量水的加入会提高萃取效果;当采用乙腈-水(84∶16, v/v)+乙腈-甲醇-水(45∶10∶45, v/v/v)时,提取效果更好,回收率为80.2%~96.1%,可以看出甲醇比例高出一定范围可能会影响*A*Ts的回收。因此,选取乙腈-水(84∶16, v/v)+乙腈-甲醇-水(45∶10∶45, v/v/v)进行提取。

**图2:**
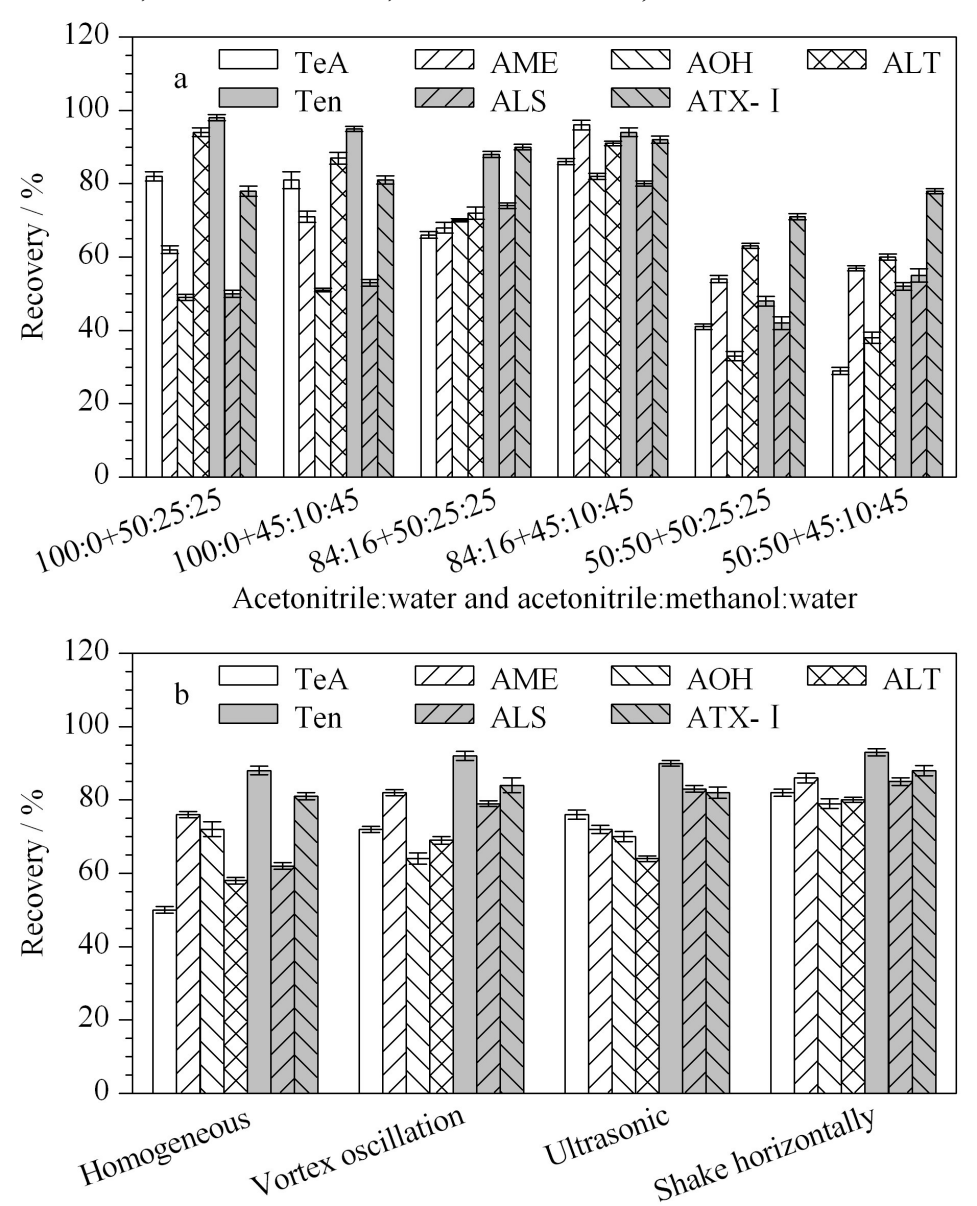
(a)提取剂比例和(b)提取方式对婴幼儿奶粉中7种链格孢霉毒素回收率的影响(*n*=5)

### 2.3 提取方式的优化

分别比较了均质(12000 r/min, 5 min)、涡旋振荡(300 r/min, 30 min)、超声波(40 ℃, 300 W, 30 min)和水平摇匀(40 ℃, 30 min)4种提取方式对7种*A*Ts回收率的影响。如图2b所示,使用均质提取时,TeA的回收率过低,仅为50.4%,这可能是因为婴幼儿奶粉成糊状,容易粘在均质器头上,造成基质损失过大,进而影响对*A*Ts的提取;涡旋振荡提取时,回收率在62.5%~91.2%之间;超声波提取时,超声时间短会导致提取*A*Ts不充分,而超声时间长又会导致奶粉样品沉积在离心管底部,致使提取剂和样品接触不充分,造成个别毒素回收率较低,7种毒素的回收率范围为63.1%~90.6%;而水平摇匀回收率好于其他3种提取方式,在79.1%~93.2%之间。Marina等^[31]^同样采取水平摇匀的方法提取婴幼儿食品中的*A*Ts,提取效果较好,检出限为0.05~1.25 μg/kg,加标回收率为83%~108%。这可能是因为在水平摇匀过程中,基质中的*A*Ts不易被破坏。

### 2.4 固相萃取条件的优化

优化固相萃取条件时,用标准溶液分别对固相萃取小柱和洗脱液条件进行优化,最后采用实际的基质样品萃取液进行确证。取一定体积的*A*Ts混合标准储备液,用乙腈稀释成50 μg/L的混合标准溶液,用于对条件的优化;在确证的基质样品中加入50 ng的*A*Ts混合标准,保持一致,对以下固相萃取条件进行系统优化^[33]^。

2.4.1 固相萃取小柱的选择

对比了HLB小柱、C_18_小柱和PSA小柱的净化效果,如图3所示,PSA小柱对TeA净化效果差,回收率仅为43.6%,推测原因是PSA填料可以去除有机酸^[34]^; HLB小柱效果略好于C_18_小柱,回收率在73.1%~92.8%之间,且HLB小柱适用性强,在上样过程中,小柱干涸不影响分离效果,易于操作^[35,36]^。因此选取HLB小柱进行净化。

**图 3:**
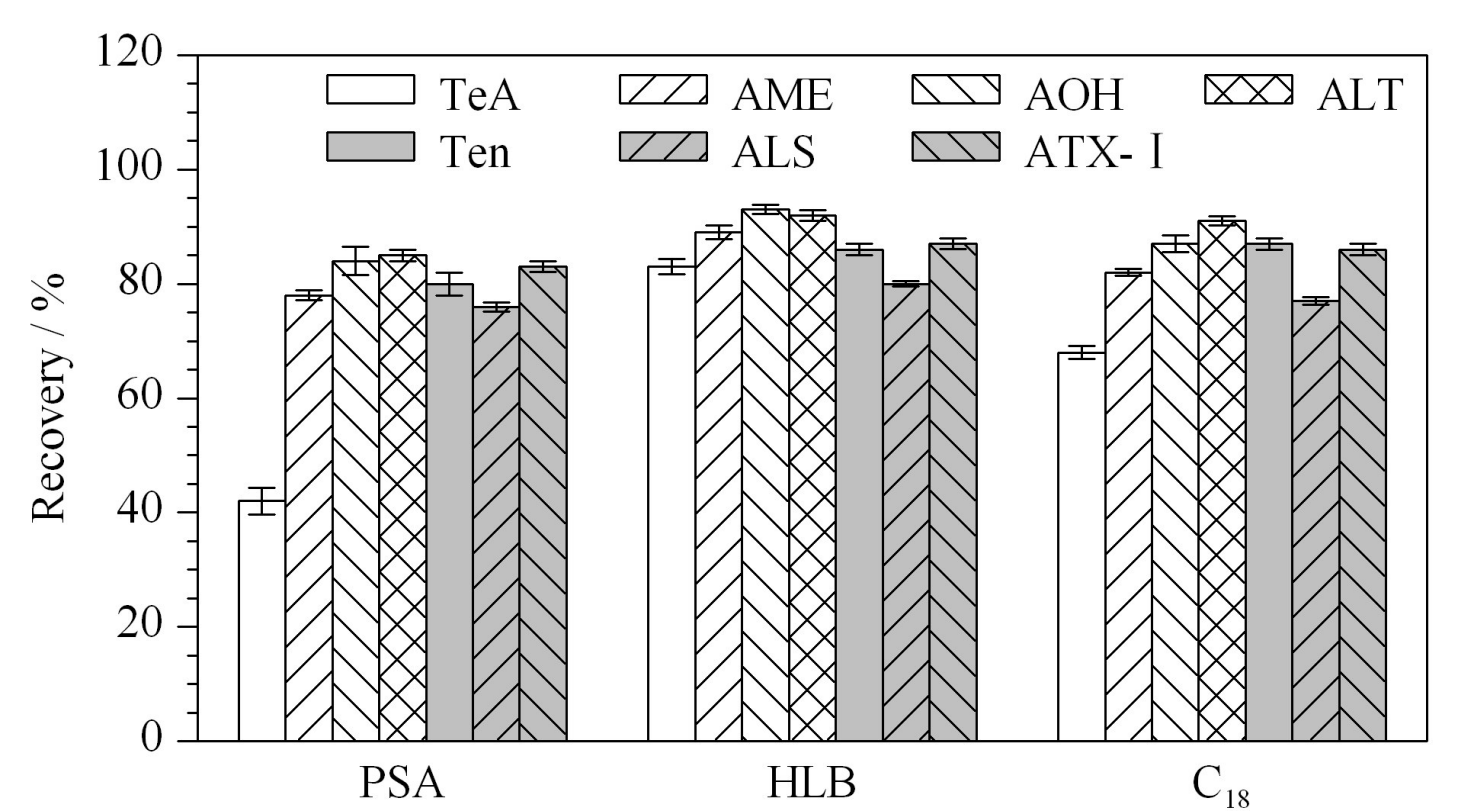
不同固相萃取小柱对7种链格孢霉毒素回收率的影响(*n*=5)

2.4.2 洗脱液种类及用量的选择

根据文献^[33]^得知,甲醇、乙腈、甲醇+乙腈洗脱小柱均在文献中被提及。由此本研究分别选用10 mL甲醇、10 mL乙腈、5 mL甲醇+5 mL乙腈作为洗脱液(如图4a),结果表明,测定后未发现明显差异。但考虑到使用甲醇时*A*Ts的回收率为76.8%~93.2%,效果略好于其他两种洗脱液,所以选取甲醇洗脱HLB小柱。考虑到洗脱液的用量可能不足以将*A*Ts完全洗脱出来,本研究分别选取了6、8、10和12 mL的甲醇洗脱液进行比较,结果如图4b所示,当甲醇添加量为6~10 mL时,7种*A*Ts的回收率均呈现上升趋势;当甲醇添加量为10 mL时,7种*A*Ts的回收率最好,在80.6%~92.2%之间;当甲醇添加量为12 mL时,7种*A*Ts的回收率变化不大,说明甲醇洗脱几乎已达到饱和。综上所述,选取10 mL甲醇为洗脱液。

**图4:**
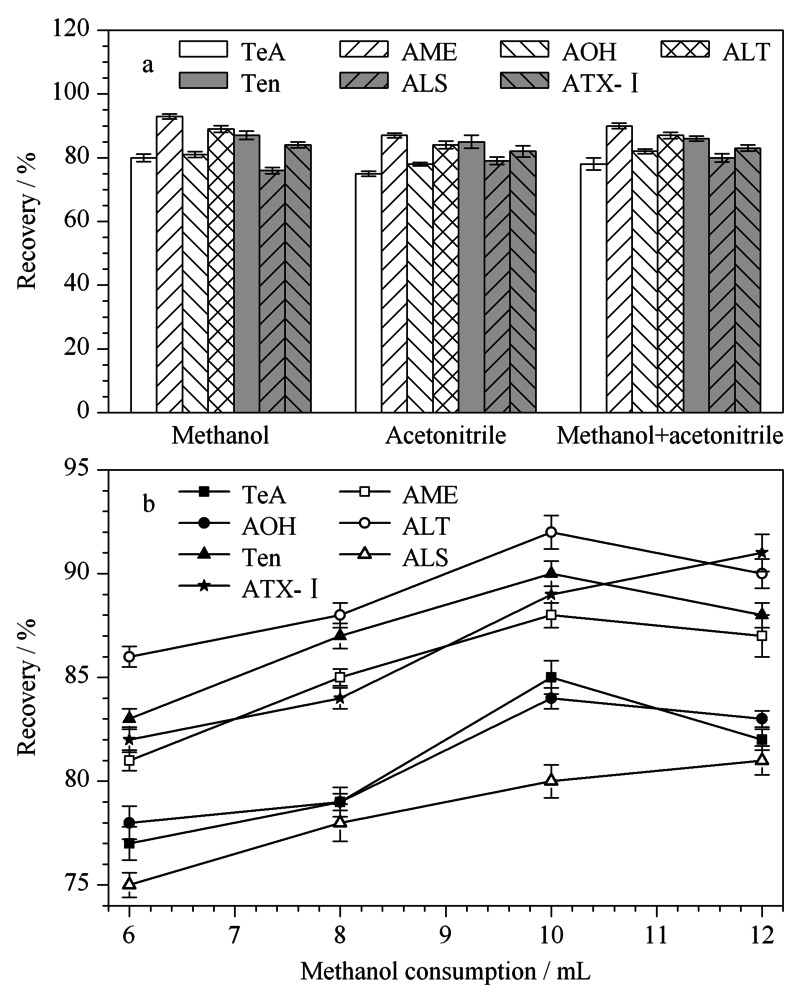
(a)洗脱液种类和(b)甲醇用量对婴幼儿奶粉中7种 链格孢霉毒素回收率的影响(*n*=5)

2.4.3 提取后水的pH的优化

因为*A*Ts需要酸化提取才会更加完全,但过度酸化会抑制回收率^[8]^,所以本研究在3次萃取之后,针对氮吹复溶的样品水溶液的pH(4、4.5、5、5.5、6)进行了考察,如图5所示,结果发现提取效果差异不是特别明显,但是pH 5.5时,*A*Ts的回收率稳定性相对较高,所以选取pH 5.5的水进行复溶,回收率为78.9%~92.3%。同时为了与其保持一致,利于上样时样品更好的萃取保留,同样选择pH 5.5的水进行活化。

**图5:**
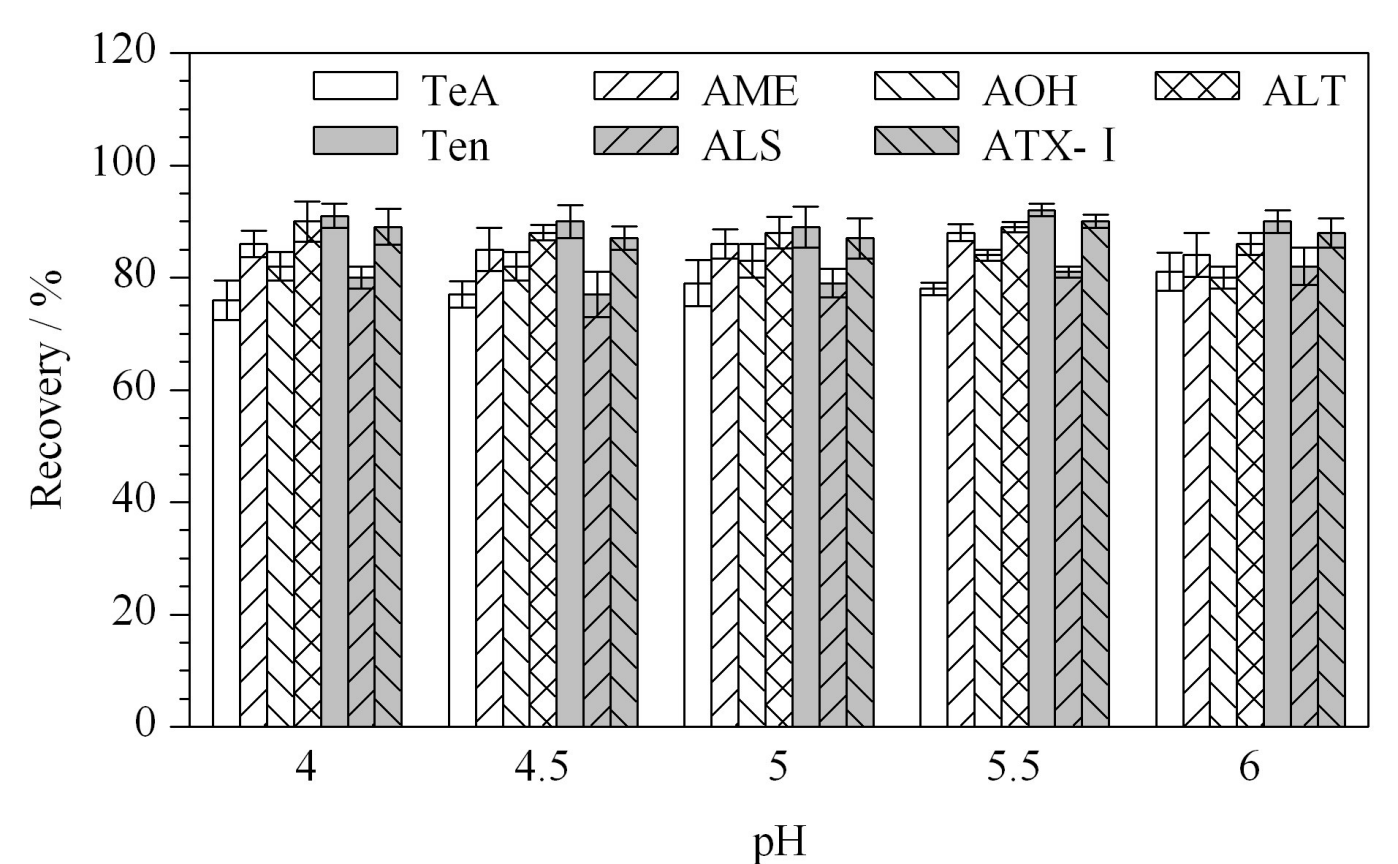
水的pH值对7种链格孢霉毒素回收率的影响(*n*=5)

2.4.4 浓缩条件的选择

实验发现,若洗脱之后在40 ℃氮吹后再次使用1 mL甲醇复溶,如图6所示,AOH、AME回收率过低,仅为20%左右,可能这2种毒素在奶粉基质中经固相萃取后再氮吹复溶会导致毒素流失,影响回收率;而不复溶时7种*A*Ts的回收率为77.4%~91.1%,即除AOH、AME外,其他毒素复溶与否无显著性差异。因此选择在经10 mL甲醇洗脱之后不浓缩,即振荡混匀后直接吸取1 mL洗脱液过0.22 μm滤膜后测定。

**图6:**
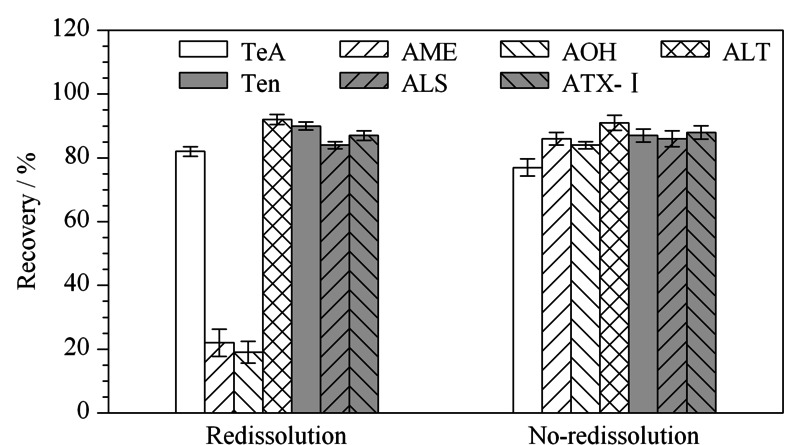
是否复溶对奶粉中7种链格孢霉毒素回收率的影响(*n*=5)

### 2.5 方法评价

2.5.1 基质效应(ME)

在UPLC-MS/MS分析中,洗脱化合物对电喷雾界面电离效率的影响表现为造成离子增强或抑制^[37]^。据报道,基质效应在UPLC-MS/MS方法分析*A*Ts时很常见^[38]^,通常通过比较标准溶液和相同浓度的阴性加标样品的响应来研究。本实验为了检测空白样品基质对目标化合物的响应值是否增强或抑制,分别用溶剂和基质空白液配制7种*A*Ts的标准混合液,质量浓度为50 μg/L,并进一步对实验结果进行比较。

根据测定的7种*A*Ts的回收率值,将ME值分为3组(低于80%、80%~120%和高于120%)。ME值在80%~120%之间为低基质效应,可以忽略。当ME值超过120%时,说明存在基质增强作用^[39]^。同时,ME值低于80%说明存在基质抑制作用。ME的计算公式如下^[40]^:

ME= $\frac{A_{2}-A_{1}}{A_{1}}$ ×100%

其中:*A*_1_为毒素标准品在特定浓度的纯溶剂(初流动相)中的平均峰面积;*A*_2_为基质空白溶液中相同浓度每种毒素标准品的平均峰面积。

如表2所示,TeA的ME值在80%~100%之间,说明TeA的基质作用较小;Ten和AOH的ME值低于80%,表现出基质抑制效应。相反AME、ALT、ALS和ATX-Ⅰ的ME值高于120%,表现出基质增强效应。根据表2所呈现的实验结果,本研究采用基质匹配的方法对基质效应进行补偿,最大程度上抵消基质效应的影响。

**表 2:** 链格孢霉毒素在婴幼儿奶粉样品基质中的基质效应

Target analyte	ME/%
TeA	91.2±2.4
AME	132.6±1.8
AOH	73.5±2.1
ALT	145.5±3.1
Ten	78.2±2.2
ALS	158.7±3.8
ATX-Ⅰ	123.4±1.2

2.5.2 线性范围与检出限

在线性关系研究中,所有标准工作溶液均在1.3节色谱-质谱条件下测定。以*x*代表质量浓度、*y*代表峰面积进行线性回归分析,用基质空白标准工作溶液做校准曲线。结果如表3所示,每种毒素在0.5~200 μg/L内都有良好的线性关系,判定系数(*R*^2^)均大于0.990。以各目标化合物信噪比(*S/N*)分别为3和10计算检出限(LOD)和定量限(LOQ), 7种*A*Ts的LOD为0.15~0.64 μg/kg,LOQ为0.54~2.24 μg/kg。

**表 3:** 婴幼儿奶粉中7种链格孢霉毒素的线性范围、线性方程、*R*^2^和检出限和定量限

Toxin	Linear range/(μg/L)	linear equation	R^2^ (n=9)	LOD/(μg/kg)	LOQ/(μg/kg)
TeA	0.5-200	y=13176.9x-1758.45	0.9958	0.64	2.24
AME	0.5-200	y=2401.37x+356.275	0.9928	0.48	1.59
AOH	0.5-200	y=2078.1x-1800.18	0.9979	0.37	1.13
ALT	0.5-200	y=39165.8x-11564.9	0.9996	0.32	1.22
Ten	0.5-200	y=14368.3x-5782.4	0.9982	0.15	0.54
ALS	0.5-200	y=4816.08x+766.478	0.9903	0.43	1.43
ATX-Ⅰ	0.5-200	y=2562.18x-3766.9	0.9945	0.21	0.73

*y*: peak area; *x*: mass concentration, μg/L.

2.5.3 样品回收率和精密度结果分析

按1.5节和1.6节的操作制备婴幼儿奶粉样品,参照GB/T 27404-2008^[41]^,分别以1倍、2倍和10倍定量限进行三水平加标试验,在一天内每个添加浓度重复进样5次,测定样品的回收率与精密度。结果如表4所示,7种*A*Ts的平均回收率为79.1%~114.3%, RSD≤8.87%。

**表 4:** 婴幼儿奶粉中7种链格孢霉毒素的添加回收率和 精密度(*n*=5)

Toxin	Spiked/(μg/kg)	Recovery/%	RSD/%
TeA	2.2	87.7	4.34
	4.4	92.6	5.10
	22.0	94.2	6.21
AME	1.6	89.9	6.33
	3.2	96.3	7.13
	16.0	114.3	5.11
AOH	1.1	86.4	4.67
	2.2	100.2	5.81
	11.0	94.1	3.68
ALT	1.2	79.1	6.54
	2.4	88.4	5.49
	12.2	82.6	7.13
Ten	0.5	93.1	4.98
	1.0	87.4	4.17
	5.0	106.1	3.16
ALS	1.4	86.6	3.69
	2.8	85.7	4.64
	14.0	80.5	5.21
ATX-Ⅰ	0.7	91.1	6.34
	1.4	93.4	5.29
	7.0	106.2	8.87

2.5.4 实际样品的测定

按照上述方法对实际婴幼儿奶粉样品(一段、二段、三段各20个样品)进行检测,结果表明,未在一段(0~6月龄)和二段(6~12月龄)奶粉中检测出真菌毒素;三段奶粉中只有1个样品检测出Ten,浓度为4.97 μg/kg。这是因为奶粉中易受污染的植物油占比较小,所以奶粉受*A*Ts的污染程度相对果蔬类产品和小麦类产品较小。而随着婴儿年龄的增长,其奶粉选用原料种类较多,被*A*Ts污染的几率也随之升高。

## 3 结论

本文采用固相萃取技术富集婴幼儿奶粉中7种*A*Ts,并结合UPLC-MS/MS,定性定量测定婴幼儿奶粉中的*A*Ts,建立了一种高效检测7种*A*Ts的检测方法。该方法操作简便,高效灵敏,快速准确,其检出限、线性关系、回收率和重现性等方法学指标均能满足*A*Ts的测定,可适用于实际样品检测。本研究开发的方法为检测更多种类的*A*Ts提供了技术支持,同时还为婴幼儿奶粉中典型链格孢霉毒素安全限量标准的制订提供理论依据,此外,本研究对于评估婴幼儿奶粉的食品安全风险、保护婴幼儿健康也具有十分重要的理论和现实意义。
